# Supplementary material for: Content-rich biological network constructed by mining PubMed abstracts
Source: BMC Bioinformatics. 2004 Oct 8;5:147. doi: 10.1186/1471-2105-5-147 (PMC528731; doi:10.1186/1471-2105-5-147)
Supplement: Additional File 5 — The original Chilibot query results of the term "long-term potentiation (LTP)" and 22 other terms, limiting the latest references analyzed to the years 1990, 1995, 2000, and 2004. [file 1471-2105-5-147-S5.bz2 › chilibotAdditionalFile5/ltp2004/html/CREB_ACTIN.html]

 


 **CREB** and **ACTIN** 
  
Found 10 abstracts in PubMed,  **10 abstracts were retrieved and analyzed**.  


---

 Search Google  |
 PDF files only 
|  EDU domain only 

---

**Interactive relationship** (e.g. stimulation, inhibition, etc)

- Immunohistochemistry revealed that  **CREB**  was mainly induced and activated in the neointimal alpha smooth muscle  **actin**  positive cells of rat carotid artery after balloon injury.  Ref: 12939230 Circulation, 2003
- Our results lead to a model of cyclin A gene regulation in which mitogens play a permissive role by stimulating early G 1 phase phosphorylation of  **CREB**  and a distinct regulatory role by cooperating with the organized  **actin**  cytoskeleton to regulate the duration of ERK signaling, the expression of cyclin D1, and the timing of pocket protein phosphorylation.  Ref: 11604497 Mol Cell Biol, 2001

- :-)

  **Parallel relationship** (e.g. studied together, co-existance, homology, etc.)

  - The induction of cyclin A is also required for S phase entry, and we now report that distinct effects of mitogens and the  **actin**  cytoskeleton on the phosphorylation of  **CREB**  and pocket proteins regulate the extent and timing of cyclin A promoter activity, respectively.  Ref: 11604497 Mol Cell Biol, 2001
  - **CREB**  phosphorylation and CRE occupancy are regulated by soluble mitogens alone, while the phosphorylation of pocket proteins requires both mitogens and the organized  **actin**  cytoskeleton.  Ref: 11604497 Mol Cell Biol, 2001
  - Distinct effects of mitogens and the  **actin**  cytoskeleton on  **CREB**  and pocket protein phosphorylation control the extent and timing of cyclin A promoter activity.  Ref: 11604497 Mol Cell Biol, 2001
